# Psychometric validation of the Angioedema Quality of Life Questionnaire (AE-QoL) for hereditary angioedema

**DOI:** 10.1186/s41687-026-01128-8

**Published:** 2026-06-19

**Authors:** Alexandra J. Feld, Martha Bayliss, Jakob B. Bjorner, Regina Rendas-Baum, Cary Thurm, Aaron Yarlas

**Affiliations:** 1 IQVIA (United States), Durham, USA; 2 Ionis Pharmaceuticals (United States), Carlsbad, USA

**Keywords:** Hereditary Angioedema (HAE), Health-Related Quality of Life (HRQoL), validity, Patient-Reported Outcomes (PROs), Psychometric validation, Rare disease outcomes

## Abstract

**Background:**

Psychometric validation of the Angioedema Quality of Life Questionnaire (AE-QoL) was conducted to determine its suitability for assessing health-related quality of life (HRQoL) in patients with hereditary angioedema (HAE).

**Methodology:**

Data were analyzed from 90 participants with HAE in OASIS-HAE (NCT05139810), a randomized, double-blind, placebo-controlled phase 3 study of donidalorsen (DAWNZERA™). Psychometric validation, assessed by post hoc analyses on scores at Baseline and Week 24 visits, included internal consistency reliability, structural and construct validity (convergent, criterion, and known-groups validity), and responsiveness.

**Results:**

Confirmatory factor analysis supported the 17-item AE-QoL measurement model (comparative fit index [CFI], Tucker-Lewis index [TLI], and standardized root mean square residual [SRMR] met thresholds). Internal consistency reliability was strong for the total, functioning, fatigue/mood, and fears/shame domains (Cronbach’s ⍺: 0.81–0.97; McDonald’s ⍵: 0.86–0.98), but weaker for the 2-item nutrition domain (Cronbach’s ⍺: 0.65–0.75; McDonald’s ⍵: 0.64–0.75). At Week 24, AE-QoL total, functioning, and fears/shame scores showed convergent validity with the 28-day Angioedema Activity Score (AAS-28) (n*r* = 0.51, 0.73, and 0.51, respectively) and with the Angioedema Control Test (AECT) (*r*=-0.77, -0.89, and − 0.70, respectively). Criterion validity was supported by moderate-to-strong correlations with HAE attack rate at Week 24 (total: *r* = 0.65; domains: *r* = 0.55–0.78, excluding fatigue/mood). Known-groups validity was demonstrated by significant differences in AE-QoL scores between patients with poor- versus well-controlled disease based on AECT score at Baseline and Week 24 (all *p* < 0.05, Cohen’s *d* effect size [ES] range: 0.63–3.65). AE-QoL total, functioning, and fears/shame scores were sensitive to changes in disease activity, with correlations’ absolute values for changes in score from Baseline to Week 24 exceeding 0.30 for AAS-28 and 0.39 for AECT, excluding fatigue/mood and nutrition domains. AE-QoL change scores for total, functioning, fears/shame, and nutrition domains effectively differentiated between subgroups with varying reductions in HAE attack frequency (all *p* < 0.02, ES range: 0.34–1.42).

**Conclusions:**

The AE-QoL demonstrated strong reliability, validity, and ability to detect change, supporting its use as an important patient-reported outcome measure for evaluating HRQoL in patients with HAE.

## Background

Hereditary angioedema (HAE) is a rare, unpredictable, debilitating disease most frequently caused by increased bradykinin levels that lead to recurrent and potentially fatal episodes of severe swelling of the skin, gastrointestinal (GI) tract, upper airway, face, and larynx, referred to as “HAE attacks” [1–3]. Attacks can range in intensity from mild (e.g., minor swelling, relatively little pain or discomfort) to severe (e.g., disfiguring, incapacitating swelling, extreme pain or discomfort), with laryngeal edema being potentially life-threatening. Symptoms typically begin during childhood or adolescence, as early as age 1, with an average age of onset of 11 years [4]. As delayed diagnosis may lead to increased morbidity with life-threatening consequences, understanding how HAE impacts children and adolescents may improve outcomes and quality of life (QoL). Prevalence estimates vary widely from 1 in 50,000 to 1 in 100,000 people globally. In the United States, the estimated prevalence of HAE is 5,860 to 6,308 (1.8–1.9 per 100,000), based on a 2021 study [2]. The true prevalence of HAE is unknown.

HAE attack frequency and severity significantly impair patients’ QoL, causing high morbidity and anxiety, pain, disfigurement, and disruption to daily life [5–10]. Banerji et al. reported that more than two-thirds of patients taking medication within the past 2 years experienced moderate-to-severe attacks, and more than three-quarters of patients reported that their most recent attack occurred within the past month [5]. Greater negative impacts on daily functioning and diminished QoL are reported by patients with higher attack frequencies [5, 11, 12]. Common triggers of HAE attacks include stress, fatigue, hormone changes, and physical trauma [3]. Since attacks are unpredictable, many people with HAE have restrictions in their daily activities, social life, and work productivity [13–15]. Even between attacks, people with HAE may have anxiety regarding their next attack, the possibility of a fatal attack, or passing HAE on to their children [5, 7, 13, 16, 17].

Given the substantial burden of HAE on QoL, accurate measurement of its impact on patients’ lives is critical, though few validated, disease-specific, patient-reported outcome (PRO) instruments are available. The Hereditary Angioedema Quality of Life Questionnaire (HAE-QoL) and the recently-developed HAE-C1INH QoL instrument were designed to assess health-related quality of life (HRQoL) in adults with HAE [18, 19]. However, these disease-specific instruments have long or mixed recall periods (the recall period for the HAE-QoL is 6 months, while the HAE-C1NH QoL uses both 7-day and 1-year recall periods), which are often not a good fit for clinical studies’ outcome measurement objectives.

The Angioedema Quality of Life Questionnaire (AE-QoL) was originally developed and validated to assess HRQoL in adults with recurrent angioedema (AE), which includes, but is not limited to, patients with HAE (19.1% of the validation sample) [20]. Recent research from Vanya et al. evaluated the relevance of the AE-QoL specifically for patients with HAE in a multinational content validation study [21]. Results from hybrid concept elicitation and cognitive debriefing (CD) interviews confirmed the relevance and clarity of AE-QoL items. Recent qualitative research, including CD of the AE-QoL in adolescent and adult patients with HAE, captured the unique emotional, social, and work- and school-related burdens experienced by these patients as well as overall endorsement of the AE-QoL, with suggestions for minor adaptations [22, 23]. Psychometric data indicated strong internal consistency reliability (⍺=0.92), test-retest reliability (intraclass correlation coefficient > 0.80), and construct validity in adults with HAE [21].

Despite the important contributions of these studies to the validity of the AE-QoL in HAE, important evidence gaps remain when evaluated against regulatory expectations [24], namely assessing the association of the AE-QoL symptom-specific criterion measures and clinical events (i.e., HAE attack rate) to support construct validity and responsiveness in adults with HAE. This study, using data from a phase 3 clinical study with numerous criterion measures and outcomes, aims to provide complementary evidence to gauge whether the AE-QoL is fit-for-purpose for assessing symptom-specific HRQoL in patients with HAE.

## Methods

### Data

This psychometric validation study utilized data from the phase 3, multinational, double-blind, randomized, placebo-controlled OASIS-HAE study (NCT05139810), which assessed the efficacy and safety of donidalorsen in adolescent and adult patients with HAE [25]. The primary endpoint was the change in time-normalized number of investigator-confirmed HAE attacks per 4 weeks (referred to as “HAE attack rate”) from Baseline (Week 0) to Week 24. Full details on the study design and protocol have been published elsewhere [25]. Briefly, patients with HAE received donidalorsen 80 mg or placebo subcutaneously once every 4 weeks or once every 8 weeks. Donidalorsen was approved by the United States Food and Drug Administration (FDA) on August 21, 2025 as a first-in-class RNA-targeted therapy for prophylaxis to prevent HAE attacks in adults and pediatric patients 12 years and older, under the brand name DAWNZERA™.

The OASIS-HAE study protocol was reviewed and approved by an independent ethics committee or institutional review board at each participating site and was carried out in accordance with Good Practice Guidelines and the Declaration of Helsinki [25]. All patients provided written informed consent to participate in the study, which was funded by Ionis Pharmaceuticals, Inc.

### Patient-reported outcome and clinical event measures

All PRO and clinical event measures included in this analysis were administered at Baseline and Week 24 study visits.

### Angioedema Quality of Life Questionnaire (AE-QoL)

The AE-QoL, developed in 2012 [20], is a 17-item, AE-specific, PRO measure from which a total score and 4 domain scores are calculated to assess the impacts of AE attacks on numerous aspects of patients’ everyday lives over the prior 4 weeks [20]. The 4 domains are:

1. **Functioning (K = 4)**: Frequency with which attacks have restricted functioning, including work, physical activity, leisure time, and social relations.
2. **Fatigue/mood (K = 5)**: Frequency of experiencing problems related to sleep (sleep disturbance, somnolence), impaired concentration, and feeling depressed.
3. **Fears/shame (K = 6)**: Frequency of experiencing emotional burden due to an attack, including fear of an unexpected attack, of increased attack frequency, and of negative long-term effects from attack treatment; shame when going out in public due to attacks; and embarrassment due to attacks.
4. **Nutrition (K = 2)**: Frequency with which attacks have restricted eating and drinking, and limited choices of food and beverages.

Item responses are scored using a 5-point Likert scale with the following answer options: “never” (0), “rarely” (1), “sometimes” (2), “often” (3), and “very often” (4), with higher scores indicating less favorable responses. Responses to all items are used to compute the total score. Raw scores for each domain and the total score are calculated and transformed into a linear scale that ranges from 0 to 100, with a score of 100 indicating the worst possible HRQoL. Established total score thresholds define levels of HRQoL impact: “no impact” (< 24), “small impact” (≥ 24 to < 39), and “moderate to large impact” (≥ 39) [26].

### Angioedema Activity Score (AAS)

The Angioedema Activity Score (AAS) is a 5-item, daily diary measure of AE attack frequency, duration, severity, and impact [27]. If no AE attack occurs during a day, the daily AAS score is 0. If 1 or more attacks occur during a day, all 5 items are rated from 1 to 3 and summed to a maximum daily total score of 15, with higher scores reflecting more frequent, severe, and impactful attacks. “AAS-28” represents the sum of daily AAS scores across a 28-day period, with scores ranging from 0 to 420 [27]. Importantly, in these analyses, the window for calculating AAS-28 scores reflects a reporting period that aligns with the AE-QoL’s 4-week recall period.

### Angioedema Control Test (AECT)

The Angioedema Control Test (AECT) is a 4-item questionnaire designed for self-reporting AE symptom control. The AECT version used in the OASIS-HAE study had a 4-week recall period, matching that of the AE-QoL. Each AECT item uses a 5-point response scale, with options ranging from 0 (“very often” or “very much”) to 4 (“not at all”) for the items measuring frequency and impact of attacks, and from 0 (“not at all”) to 4 (“very well”) on the item measuring treatment control [28]. A total score is calculated as the sum of all 4 items, ranging from 0 to 16, with higher scores indicating more well-controlled disease. Validation of this instrument has identified a cut-point of 10 as indicative of disease control, such that an AECT score ≥ 10 indicates well-controlled disease, and a score < 10 indicates poor disease control [29].

### Monthly investigator-confirmed hereditary angioedema attack rate

Computation of the HAE attack rate involved 3 steps. First, patients reported attacks to their physician (study investigator) within 72 h of onset. To optimize accuracy of reporting, patients were also prompted to report any HAE attacks during weekly study contact visits, and descriptive data were captured electronically via the daily AAS [27] questionnaire entries, as noted above. Investigator-confirmed HAE attacks were defined as events, separated by 24-hour symptom-free intervals, and characterized by signs or symptoms consistent with an attack in at least 1 pre-specified bodily location. Detailed information was collected about timing, location, severity, description, course, impact of signs and symptoms, and treatment.

Next, the clinician reviewed the data collected to confirm whether reported attack symptoms were indicative of an HAE attack, per the clinical study protocol.

Finally, the monthly investigator-confirmed HAE attack rate was calculated as the number of confirmed attacks occurring during each 4-week interval, with intervals beginning on study Day 1. These data were captured in the AAS-28. Thus, the monthly investigator-confirmed HAE attack rate was calculated every 4 weeks from Baseline through the 24-week treatment period. For analysis at Baseline and Week 24, the 4-week attack rate timeframe aligns with the AE-QoL recall period.

### Psychometric analyses

All psychometric analyses were post hoc and used blinded data pooled across all treatment arms at Baseline and Week 24 (combined placebo group and the 2 active treatment arms, *N* = 90). Accordingly, no formal multiplicity adjustment was applied. Demographic and baseline characteristics of the sample were described, along with the number and proportion of patients with evaluable PRO scores at Baseline and Week 24.

Data analyses were performed using SAS Version 9.4 (SAS Institute Inc., Cary, NC, USA), except where stated otherwise.

### Measurement model

The AE-QoL’s measurement model supporting 4 domain scores and a total score has not been previously studied in patients with HAE. Using confirmatory factor analysis (CFA), 2 models were tested at Baseline: (1) a correlated 4-factor model, as proposed by Weller et al. [20]; and (2) a bifactor model [30] including a general factor to represent the use of a total score and 4 factors capturing variation associated with distinct groups of items.

This factor analytic approach aimed to test the validity and relative value of the AE-QoL’s 4-factor structure, as well as to evaluate the validity of using the total score as a global measure [31], an assessment that was made by calculating the explained common variance, defined as the proportion of common variance attributable to the general factor [32].

Model fit was evaluated at Baseline using pre-specified fit criteria, including the comparative fit index (CFI) and Tucker-Lewis index (TLI) (both ≥ 0.90); standardized root mean square residual (SRMR) ≤ 0.08 [33]; and root mean square error of approximation (RMSEA) ≤ 0.08 [34] to indicate an acceptable fit. RMSEA values > 0.1 were interpreted as indicating unacceptable fit [35]. Model assessment considered recent research that indicated consensus toward triangulating across goodness-of-fit indices [34].

CFA was conducted in Mplus Version 8 [36] using weighted least squares mean- and variance-adjusted estimation, recommended for modeling ordered categorical response options [37]. Items with standardized loadings < 0.30 on their assigned factor were considered indicative of potential measurement model misspecification.

### Scale distribution analyses

Floor and ceiling effects were examined for total and domain scores using an a priori threshold of ≥ 15%, calculated as the percentage of participants scoring the minimum and maximum possible scores on each scale [38].

### Internal consistency reliability

Internal consistency reliability of the AE-QoL total and domain scores based on Cronbach’s alpha (⍺) and McDonald’s omega (⍵) was measured to evaluate the reliability of the AE-QoL. Adequate internal consistency reliability was indicated by reliability coefficients ≥ 0.70 [39] using Baseline and Week 24 visit data.

### Construct validity

Construct validity was analyzed via convergent, criterion, and known-groups validity.

### Convergent validity

Pearson correlations (*r*), where |*r*| ≥ 0.40 supported convergent validity [40], assessed the extent to which AE-QoL total and domain scores were associated with AAS-28 and AECT scores at Baseline and Week 24. Positive correlations between AE-QoL and AAS-28 were hypothesized a priori, since higher scores on both measures indicate worse outcomes related to HAE attacks. AECT scores were hypothesized a priori to be negatively correlated with AE-QoL total and domain scores, as lower scores indicate poorer disease control.

### Criterion validity

Pearson correlations, where |*r*| ≥ 0.40 between assessments of AE-QoL total and domain scores and monthly investigator-confirmed HAE attack rates over the 4-week period preceding the study visit, was considered evidence supporting adequate criterion validity [41]. It was hypothesized a priori that positive correlations would be observed between AE-QoL scores and monthly investigator-confirmed HAE attack rate, as higher numbers for each indicate worse health outcomes. Analyses were performed at Baseline and Week 24.

### Known-groups validity

Known-groups validity was assessed by comparing mean AE-QoL total and domain scores among patients with AECT scores < 10 (poor disease control) and AECT scores ≥ 10 (well-controlled disease), using independent-samples *t*-tests and Cohen’s *d* effect sizes (ES) for standardized mean differences. For Cohen’s *d*, a value of < 0.2 is interpreted as a negligible effect, ≥ 0.2 and < 0.5 as a small effect, ≥ 0.5 and < 0.8 as a medium-sized effect, and ≥ 0.8 as a large effect [42]. Given that greater HRQoL impairment due to HAE attacks is expected among patients with more poorly controlled disease, AE-QoL scores were hypothesized a priori to be higher (i.e., worse) than those of patients with well-controlled disease. Analyses were performed at Baseline, where most patients were expected to have poor disease control, and at Week 24, where most patients were expected to have well-controlled disease.

### Ability to detect change

#### Correlational analyses

Pearson correlations, where |*r*| ≥ 0.30 was considered supportive of the instrument’s ability to detect change adequately [43], were evaluated between AE-QoL total and domain score changes and changes in AAS-28 and AECT scores from Baseline to Week 24. It was hypothesized a priori that positive correlations would be observed between changes in AE-QoL scores and changes in AAS-28 scores over time, and negative correlations would be observed between changes in AE-QoL scores and changes in AECT scores over time.

#### Analyses based on group differences

The ability to detect change was also evaluated by comparing the magnitudes of changes in mean AE-QoL scores for patients who experienced reduction in monthly investigator-confirmed HAE attack rates of different magnitudes: reduction of less than 50%, reduction of at least 50% but below 90%, and reduction of at least 90%. These groupings supported study endpoints based on HAE attack rate. Differences in means across groups were tested using analysis of variance (ANOVA), with magnitude of mean differences for pairwise comparisons expressed using Cohen’s *d* ES.

## Results

### Sample characteristics and descriptive statistics

Data from 90 patients with HAE were included in the analytic sample; 48 (53%) were female and 79 (88%) were Non-Hispanic White, with a mean age of 37 years old (range: 12–68 years old, with 7 patients under 18 years of age and 55 over the age of 30; Table 1). Mean AE-QoL total scores at Baseline indicated a moderate-to-large impact of HAE attacks on HRQoL (mean [SD] = 46.0 [17.3]; Table 2) and a small impact of attacks on HRQoL at Week 24 (25.1 [20.1]) [26]. At Baseline, mean (SD) domain scores ranged from 34.6 (23.3) for nutrition to 54.6 (22.2) for fears/shame. At Week 24, mean (SD) scores ranged from 16.3 (24.4) for functioning to 30.0 (27.2) for fears/shame.

**Table 1:** Demographic characteristics of patients with HAE (*N* = 90)

Patient Characteristics	*n* (%)
**Sex**	
Female	48 (53.3)
Male	42 (46.7)
**Age (years)**	
Mean (SD)	37.2 (13.88)
Min, Max	12, 68
**Age Group (years)**	
12–14	3 (3.3)
15–17	4 (4.4)
18–20	3 (3.3)
21–30	25 (27.8)
31–50	39 (43.3)
51+	16 (17.8)
**Race/Ethnicity**	
Non-Hispanic White	79 (87.8)
Hispanic/Latino	6 (6.7)
Non-Hispanic Other	5 (5.6)

Abbreviations: HAE, hereditary angioedema; Max, maximum observed value; Min, minimum observed value; SD, standard deviation

**Table 2:** Descriptive statistics for AE-QOL total and domain scores at baseline and week 24

AE-QoL Domain	*n*	Mean	SD	Median	IQR (Q1, Q3)	Min	Max	Floor (%)	Ceiling (%)
**Baseline**									
Total	90	46.0	17.3	47.8	35.3, 55.9	4.4	80.9	0.0	0.0
Functioning	90	47.0	22.4	45.4	31.3, 68.8	0.0	100	2.2	1.1
Fatigue/mood	90	39.4	20.1	40.0	25.0, 55.0	0.0	85.0	4.4	0.0
Fears/shame	90	54.6	22.2	54.4	41.7, 70.8	4.2	100	0.0	2.2
Nutrition	90	34.6	23.3	29.8	12.5, 50.0	0.0	100	8.9	2.2
**Week 24**									
Total	82	25.1	20.1	19.1	10.3, 39.7	0.0	77.9	8.5	0.0
Functioning	82	16.3	24.4	0.0	0.0, 25.0	0.0	93.8	56.1	0.0
Fatigue/mood	82	29.6	21.6	30.0	10.0, 50.0	0.0	75.0	20.7	0.0
Fears/shame	82	30.0	27.2	20.8	8.3, 54.2	0.0	95.8	15.9	0.0
Nutrition	82	16.6	20.9	12.5	0.0, 25.0	0.0	100	43.9	1.2

Abbreviations: AE-QoL, Angioedema Quality of Life Questionnaire; IQR, interquartile range; Max, maximum observed value; Min, minimum observed value; Q1, first quartile; Q3, third quartile; SD, standard deviation

### Measurement model and confirmatory factor analysis

In both the 4-factor and bifactor models, CFI, TLI, and SRMR were at or better than the recommended minimum values indicating acceptable fit (Table 3). While RMSEA for the 4-factor model (0.095) exceeded the recommended threshold associated with acceptable fit, it remained below 0.10. The bifactor model had an RMSEA suggesting acceptable fit (0.079). Overall, triangulating across indices [34] generated a positive assessment of both measurement models for 3 or 4 of the 4 pre-specified goodness-of-fit indices.

**Table 3:** Global fit statistics and standardized factor loadings for AE-QoL 4-factor and bifactor CFA baseline models

**Global Fit Statistic**		**4-Factor Model**		**Bifactor Model**			
**Chi-Square (df)**		202.10 (113)		158.71 (103)			
***p*** **-value**		< 0.001		< 0.001			
**CFI**		0.955		0.972			
**TLI**		0.946		0.963			
**SRMR**		0.070		0.061			
**RMSEA (90% CI)**		0.095 (0.074, 0.116)		0.079 (0.053, 0.102)			
**Factor**	**Item Content**	**Correlated Model Loadings**		**General Factor Loadings**		**Specific Factor Loadings**	
		**Est**	**SE**	**Est**	**SE**	**Est**	**SE**
**Functioning**	Restrictions on work	0.88	0.04	0.77	0.06	0.39	0.08
	Restrictions on physical activities	0.82	0.04	0.68	0.07	0.47	0.08
	Restrictions on leisure time	0.87	0.03	0.73	0.06	0.49	0.08
	Restrictions on social relations	0.92	0.03	0.77	0.06	0.50	0.09
**Fatigue/mood**	Difficulty falling asleep	0.76	0.05	0.50	0.09	0.59	0.09
	Waking up during the night	0.73	0.06	0.44	0.10	0.64	0.08
	Feeling tired during the day	0.87	0.04	0.58	0.09	0.64	0.09
	Difficulty concentrating	0.69	0.07	0.47	0.09	0.51	0.09
	Feeling depressed	0.68	0.07	0.53	0.08	0.33	0.09
**Fears/shame**	Burden due to AE episode	0.83	0.04	0.78	0.06	0.18	0.08
	Fear of sudden AE episode	0.83	0.04	0.71	0.06	0.42	0.08
	Fear of increased frequency of AE episodes	0.87	0.03	0.78	0.06	0.38	0.10
	Ashamed to go out in public because of AE	0.78	0.05	0.48	0.10	0.72	0.08
	Feel embarrassed/self-conscious about AE	0.76	0.05	0.42	0.10	0.81	0.10
	Fear long-term effects of treatment	0.53	0.07	0.51	0.07	0.05	0.10
**Nutrition**	Restrictions on eating/drinking	0.90	0.10	0.61	0.07	0.53	0.06
	Limited choice of foods and beverages	0.59	0.09	0.40	0.10	0.53	0.06

Abbreviations: AE, angioedema; AE-QoL, Angioedema Quality of Life Questionnaire; CFA, confirmatory factor analysis; CFI, comparative fit index; CI, confidence interval; df, degrees of freedom; Est, estimate; RMSEA, root mean square error of approximation; SE, standard error; SRMR, standardized root mean square residual; TLI, Tucker-Lewis index; WLSMV, weighted least squares mean- and variance-adjusted

Note: All models used WLSMV estimation with pooled data from across available timepoints.^a^ Explained common variance for the general factor = 0.59.

The general factor in the bifactor model explained 59% of the common variance present in the data captured by the AE-QoL, showing the importance of the total score (Table 3). Items with stronger loadings on the specific factors than on the general factor included items 1 through 4 for the fatigue/mood domain and items 4 and 5 for the fears/shame domain. Together, CFA findings suggest the importance of both the total and the domain scores in this patient population.

### Scale distribution analyses

At Baseline, floor and ceiling effects were not observed for total or domain scores. At Week 24, significant floor effects were observed for all domains (range: 15.9% for fears/shame to 56.1% for functioning; Table 2) but not for the total score.

### Internal consistency reliability

The AE-QoL showed excellent internal consistency reliability for the total score, with a Cronbach’s ⍺ of 0.90 and 0.94 and a McDonald’s ⍵ of 0.93 and 0.97 at Baseline and Week 24, respectively (Table 4). For AE-QoL domains, internal consistency reliability was adequate (≥ 0.70) to excellent (≥ 0.90) across study visits, with the only exceptions being for the nutrition domain, where both Baseline and Week 24 estimates were below the recommended threshold of 0.70 (both ≥ 0.64).

**Table 4:** Internal consistency reliability for AE-QoL total and domain scores at baseline and week 24

AE-QoL Domain	Number of Items	Baseline		Week 24	
		⍺	⍵	⍺	⍵
Total	17	0.90	0.93	0.94	0.97
Functioning	4	0.90	0.91	0.97	0.98
Fatigue/mood	5	0.81	0.86	0.87	0.90
Fears/shame	6	0.83	0.95	0.92	0.96
Nutrition	2	0.65	0.64	0.75	0.75

Abbreviation: AE-QoL, Angioedema Quality of Life Questionnaire

⍺, Cronbach´s alpha and ⍵, McDonald’s omega are measures of internal consistency reliability

Note: Adequate internal consistency reliability indicated by reliability coefficients ≥ 0.70 [35]

### Construct validity

### Convergent validity

Table 5 provides Pearson correlation coefficients between AE-QoL scores and AAS-28 and AECT scores at Baseline and Week 24. As hypothesized, AE-QoL total scores adequately correlated in a positive direction with AAS-28 at Week 24 (*r* = 0.51) and were nearly (*r* = 0.39) at the recommended threshold of 0.40 at Baseline. The functioning domain scores showed adequate-to-strong convergence with AAS-28 scores: *r* = 0.41 at Baseline and *r* = 0.73 at Week 24. There was also adequate convergence between fears/shame domain scores and AAS-28 at Week 24 (*r* = 0.51). Correlations between AAS-28 and fatigue/mood or nutrition domain scores did not exceed the recommended threshold at Baseline or Week 24.

**Table 5:** Correlations between AE-QoL scores and AAS-28 and AECT scores at baseline and week 24

AE-QoL Domain	AAS-28		AECT	
	Baseline (*n* = 62)	Week 24 (*n* = 62)	Baseline (*n* = 89)	Week 24 (*n* = 85)
Total	0.39	**0.51**	**-0.59**	**-0.77**
Functioning	**0.41**	**0.73**	**-0.62**	**-0.89**
Fatigue/mood	0.33	0.04	-0.32	-0.33
Fears/shame	0.24	**0.51**	**-0.53**	**-0.70**
Nutrition	0.26	0.33	-0.32	**-0.64**

Abbreviations: AAS-28, Angioedema Activity Score – 28 days; AECT, Angioedema Control Test; AE-QoL, Angioedema Quality of Life Questionnaire

Note: Bolded values indicate Pearson correlations |*r*| ≥ 0.40

As hypothesized, AE-QoL total and domain scores had strong negative correlations with AECT at Week 24 (all *r *≤ -0.64), apart from the fatigue/mood domain (*r *= -0.33). At Baseline, AE-QoL total, functioning, and fears/shame scores adequately correlated with AECT (all *r *≤ -0.53); however, nutrition and fatigue/mood domain scores (both *r *= -0.32) did not exceed the |0.40| threshold.

### Criterion validity

As hypothesized, AE-QoL total scores had strong positive correlations with HAE attack rate (*r* = 0.65) at Week 24, indicating good criterion validity (Table 6). In addition, all domain scores except for fatigue/mood had moderate-to-strong correlations with HAE attack rate at Week 24 (correlations range: 0.55 to 0.78), supporting criterion validity for functioning, fears/shame, and nutrition domains. At Baseline, correlations did not exceed the |0.40| threshold.

**Table 6:** Criterion validity correlations between AE-QoL scores and HAE attack rate at baseline and week 24

AE-QoL Domain	Baseline (*n* = 90)	Week 24 (*n* = 81)
Total	0.34	**0.65**
Functioning	0.31	**0.78**
Fatigue/mood	0.32	0.30
Fears/shame	0.18	**0.55**
Nutrition	0.35	**0.57**

Abbreviations: AE-QoL, Angioedema Quality of Life Questionnaire; HAE, hereditary angioedema

Note: Bolded values indicate Pearson correlations |*r*| ≥0.40

### Known-groups validity

As hypothesized, at both Baseline and Week 24 visits, all AE-QoL total and domain mean scores were significantly higher in patients with poor disease control (AECT score < 10) compared to those with well-controlled disease (AECT score ≥ 10; total scores: all *p* < 0.001; domains: all *p* < 0.02; Table 7). ES for pairwise differences were very large for AE-QoL total scores at Baseline and Week 24 (ES = 1.26 and 2.50, respectively). In addition, apart from fatigue/mood at Baseline and Week 24 and nutrition domain scores at Baseline, where medium-sized differences were observed (ES range: 0.63 to 0.76), AE-QoL domain mean score differences were very large (ES range: 1.16 to 3.65). 

**Table 7:** Mean differences in AE-QoL scores by AECT disease control subgroups at baseline and week 24

AE-QoL Domain	Poor Disease Control (AECT < 10)		Well-Controlled Disease (AECT ≥ 10)		*p*-value*	ES, Cohen’s *d*
	n	Mean (SD)	n	Mean (SD)		
**Baseline**						
Total	69	50.3 (15.9)	20	30.9 (13.4)	**< 0.001**	1.26
Functioning	69	52.5 (21.0)	20	28.0 (17.2)	**< 0.001**	1.21
Fatigue/mood	69	42.2 (20.4)	20	29.7 (16.6)	**0.015**	0.63
Fears/shame	69	59.8 (19.9)	20	36.5 (21.3)	**< 0.001**	1.16
Nutrition	69	38.0 (24.1)	20	23.0 (16.7)	**0.011**	0.66
**Week 24**						
Total	18	52.3 (14.8)	64	17.5 (13.7)	**< 0.001**	2.50
Functioning	18	54.5 (21.0)	64	5.6 (10.5)	**< 0.001**	3.65
Fatigue/mood	18	41.9 (19.2)	64	26.2 (21.1)	**0.006**	0.76
Fears/shame	18	63.0 (20.4)	64	20.8 (20.8)	**< 0.001**	2.03
Nutrition	18	41.7 (25.4)	64	9.6 (12.6)	**< 0.001**	1.99

* *p-*values are based on an independent-samples *t*-test comparing the mean AE-QoL scores between the two disease control subgroups

Abbreviations: AECT, Angioedema Control Test; AE-QoL, Angioedema Quality of Life Questionnaire; ES, effect sizes; SD, standard deviation

Note: Bolded values indicate statistical significance at *p*-value <0.05

At Week 24, the mean AE-QoL total score among patients with poor disease control indicated moderate-to-large HRQoL impact (mean [SD] = 52.3 [14.8]), whereas the mean total score among patients with well-controlled disease indicated no HRQoL impact (mean [SD] = 17.5 [13.7]). Together, these results support known-groups validity of AE-QoL in patients with HAE.

### Ability to detect change

### Correlational analyses

Change from Baseline to Week 24 in AE-QoL total and functioning and fears/shame domain scores, showed good ability to detect change in AAS-28 (all *r* ≥ 0.30; Table 7) in the hypothesized direction.

**Table 8:** Correlations between changes in AE-QoL and AAS-28 and AECT scores from baseline to week 24

AE-QoL Domain	AAS-28 (*n* = 49)	AECT (*n* = 79)
Total	**0.40**	**-0.67**
Functioning	**0.39**	**-0.73**
Fatigue/mood	0.21	-0.28
Fears/shame	**0.30**	**-0.61**
Nutrition	0.26	**-0.39**

Abbreviations: AAS-28, Angioedema Activity Score – 28 days; AECT, Angioedema Control Test; AE-QoL, Angioedema Quality of Life Questionnaire

Note: Bolded values indicate Pearson correlations |*r*| ≥ 0.30

Furthermore, change in AE-QoL total and all domain scores showed good ability to detect change in AECT scores from Baseline to Week 24 (correlations range: -0.39 to -0.73; Table 7), excluding the fatigue/mood domain (*r *= -0.28).

### Analyses based on group differences

At Week 24, the change in AE-QoL total and functioning, fears/shame, and nutrition domain scores were able to differentiate between reductions in HAE attack rate subgroups (all *p* < 0.02; Table 8). The magnitude of differences indicated medium-to-large effects for AE-QoL total and functioning scores for comparisons between the < 50% versus 50%-90% reduction groups and the 50%-90% versus ≥ 90% reduction groups (ES range: 0.63 to 1.06; Table 8), further supporting the responsiveness of the measure.

**Table 9:** Mean differences in AE-QoL scores between HAE attack rate subgroups at week 24

AE-QoL Domain	Reductions in HAE Attack Rate, Mean (SD)			*p*-value*	ES, Cohen’s *d*	
	< 50% (n=15)	≥ 50% and < 90% (n=12)	≥ 90% (n=54)		< 50% vs. ≥ 50% and < 90%	≥ 50% and < 90% vs. ≥ 90%
Total	-0.3 (12.7)	-16.1 (17.2)	-26.9 (17.2)	**< 0.001**	1.06	0.63
Functioning	0.8 (23.8)	-18.5 (19.9)	-40.6 (22.6)	**< 0.001**	0.87	1.00
Fatigue/mood	-1.3 (13.2)	-7.0 (29.1)	-12.0 (21.7)	0.230	0.26	0.22
Fears/shame	1.1 (16.4)	-23.3 (18.2)	-31.9 (23.6)	**< 0.001**	1.42	0.38
Nutrition	-4.2 (25.3)	-12.3 (22.0)	-21.9 (20.7)	**0.018**	0.34	0.46

* *p-*value is based on ANOVA model comparing mean AE-QoL scores between the three HAE attack rate reduction subgroups

Abbreviations: AE-QoL, Angioedema Quality of Life Questionnaire; ANOVA, analysis of variance; ES, effect sizes; HAE, hereditary angioedema; SD, standard deviation

Note: Bolded values indicate statistical significance at *p*-value < 0.05

## Discussion

The AE-QoL is the most frequently-used tool to capture HRQoL in HAE clinical studies [25, 44–46] and has been identified as the most appropriate tool for this purpose in clinical studies of patients with HAE [21]. Initially developed and validated with a small number of patients with HAE within a larger sample of patients with recurrent AE [20, 47], previous evaluation of its psychometric properties in HAE has focused on concept saturation, content validity, and limited measurement properties [21–23]. This study sought to evaluate the measurement properties of the AE-QoL, incorporating both other patient-reported symptom-specific criterion measures (AAS-28, AECT) and clinical events (investigator-confirmed attack rates).

These results provide overall support for good psychometric properties of the AE-QoL in patients with HAE (with the exception of the fatigue/mood domain), with findings strengthened by the consistent 4-week recall period across all instruments and measures. Reliability of the AE-QoL was supported for the total, functioning, fatigue/mood, and fears/shame domains, as both Cronbach’s ⍺ and McDonald’s ⍵ demonstrated good to excellent levels of reliability. For the 2-item nutrition domain, reliability was low (0.65) at Baseline but adequate (0.75) at follow-up. The internal consistency findings agree with those by Can et al. [48], where the nutrition domain underperformed (Cronbach’s ⍺ = 0.55) but the remaining domains had excellent internal consistency reliability (Cronbach’s ⍺ ≥ 0.88). Construct validity was demonstrated via convergent, criterion, and known-groups validity. Generally, support for construct validity was stronger at Week 24 than at Baseline, where patients had high disease activity and less disease control. Increased heterogeneity following treatment-related improvements may have resulted in stronger correlations at Week 24. Convergent validity showed adequate-to-strong correlations with the AAS-28 and AECT both for AE-QoL total score and functioning and fears/shame domain scores. Strong correlations of AE-QoL total score with HAE attack rate, and moderate-to-strong correlations of functioning, fears/shame, and nutrition domains support the criterion validity of the tool. Here also, our findings resemble the overall trends reported by Weller et al. and Morioke et al., where correlations between the Dermatology Life Quality Index and AE-QoL domains were typically largest for the AE-QoL functioning domain and smallest for the AE-QoL nutrition domain [20, 49]. In the current study, the AE-QoL also demonstrated strong known-groups validity with AECT. Mendivil et al. [17] reported large to medium-sized differences across HAE attack frequency-based subgroups for all (effect sizes: -0.89 to -0.41) except the nutrition domain (effect size = -0.20). Despite high floor effects for domain (but not total) scores being observed at Week 24, suggesting shortcomings in the assessment of patients with better HRQoL, ability to detect change (i.e., responsiveness) was demonstrated both via correlational analyses with AAS-28 and AECT, and via analyses based on group differences with HAE attack rate subgroups. These results agree with those reported by previous studies [44, 47, 48], where consistently moderate associations between changes in AE-QoL scores and changes in measures of disease activity, as well as associations between change in AE-QoL scores and change in frequency/rate of AE episodes, were reported. The current findings provide critical evidence needed to support and validate the suitability of the AE-QoL to measure HRQoL and treatment benefit specifically in patients with HAE as had been reported in clinically related, but distinct, patient groups. In CFA analyses, the RMSEA for the 4-factor model did not meet the 0.08 threshold for good fit, but CFI, TLI, and SRMR all supported the hypothesized 4-factor structure. In the bifactor model, all goodness-of-fit indices supported the factor structure, with 59% of the common variance explained by the global factor, although relevant domain-specific loadings were found, particularly for fatigue/mood, nutrition, and 2 items (on embarrassment) from the fears/shame domain. This suggests that the total score does not summarize all item information and that unique information is provided by domain scores. However, the comparatively worse performance of the fatigue/mood domain with regards to convergent validity, criterion validity, known groups validity, and ability to detect change suggests that the unique information for the fatigue/mood domain may not be clinically relevant. From a content validity perspective, the domain lacks conceptual cohesion, as its 5 items encompass 3 distinct, though related, constructs: fatigue and sleep (e.g., “feeling tired during the day” and “waking up during the night”), mood (“feeling depressed”), and cognition (“difficulty concentrating”). Further, the way in which items aggregate may be dependent on the specific patient profile. Indeed, fatigue, sleep disturbance, mood changes, and cognitive difficulties, do not occur uniformly or concurrently across patients as prodromal manifestations in HAE [50, 51]. While individual patients may report consistent personal patterns, there is substantial variability in their simultaneous occurrence. Thus, it is likely that aggregating these distinct concepts into a single score results in weaker psychometric results. Findings from the CFA suggest that the AE-QoL domains are not equally related to the total score, highlighting that some constructs contribute more strongly to overall HRQoL than others.

This study has several strengths, including matching recall periods for all instruments and a sample size of 90, which, although relatively small for psychometric analyses, is larger relative to previous AE-QoL validation studies in HAE samples [21]. Additionally, the use of symptom-specific PRO measures and criterion clinical events (i.e., HAE attack rate) to evaluate the construct validity of the AE-QoL, the inclusion of factor analysis to test the validity and relative value of the 4-factor structure, as well as to evaluate the validity of the AE-QoL total score as a global measure, and the first known evaluation of the instrument’s ability to detect change in patients with HAE, are all particular strengths that both supplement and expand upon previous validation work. Together, these strengths provide new evidence in psychometric validation of the AE-QoL. Limitations of this study include a population sourced from a clinical study, which may not provide a comprehensive representation of the broader HAE patient population; therefore, the generalizability of results beyond the study population is unknown. Of note, while emotional and functioning impacts resulting from high attack frequency and pharmacophobia have been documented in the literature [52, 53], these impacts may not be captured within the close monitoring setting of a clinical study. Because analyses were post hoc and exploratory, findings should be considered hypothesis-generating rather than confirmatory, and further research would strengthen the evidence of the observed patterns. Furthermore, the analyses were unable to explore test-retest reliability due to timing of assessments, and divergent validity was not evaluated due to lack of suitable variables. Additionally, constraints to statistical power that are common across studies in rare disease populations may also limit findings. Finally, while this study is among the first to include adolescents with HAE in psychometric analyses of the AE-QoL, the limited sample size precluded subgroup analyses and may be insufficient to adequately power the factor analyses done to assess structural validity. Further research is needed, with larger cohorts, to validate the AE-QoL across diverse groups of adolescents with HAE and consolidate findings of structural validity.

## Conclusions

Supported by analyses of data from a phase 3 clinical study and building upon previous assessment of the psychometric validity of the AE-QoL in patients with HAE, this study affirms the AE-QoL to be a reliable, valid, and appropriate instrument for assessing HRQoL in patients with HAE. By evaluating the AE-QoL against symptom-specific PRO measures and criterion clinical events, this analysis supports its psychometric validity by demonstrating reliability, construct validity, and ability to detect change in patients with HAE.

## Data Availability

Data requests from qualified researchers to the corresponding author will be considered once all three of the following criteria are met: (1) 12 months from marketing approval of the study drug in both the United States and European Union; (2) 18 months from the conclusion of the study; and (3) 6 months from publication of this article.

## Abbreviations

AAS: Angioedema Activity Score
AE: Angioedema
AE-QoL: Angioedema Quality of Life Questionnaire
AECT: Angioedema Control Test
ANOVA: Analysis of variance
CD: Cognitive debriefing
CFA: Confirmatory factor analysis
CFI: Comparative fit index
CI: Confidence interval
df: Degrees of freedom
ES: Effect sizes
Est: Estimate
FDA: United States Food and Drug Administration
GI: Gastrointestina
HAE: Hereditary angioedema
HAE-QoL: Hereditary Angioedema Quality of Life Questionnaire
HRQoL: Health-related quality of life
IQR: Interquartile range
NCT: ClinicalTrials.gov identifier
PRO: Patient-reported outcome
QoL: Quality of life
RMSEA: Root mean square error of approximation
SD: Standard deviation
SE: Standard error
SRMR: Standardized root mean square residual
TLI: Tucker–Lewis index
WLSMV: Weighted least squares mean- and variance-adjusted
